# Fluorescence HPLC Analysis of Teriflunomide in Human Plasma Following Derivatization with 4-Chloro-7-Nitrobenzofurazan: Method Development and Application to a Prototype Pharmacokinetic Evaluation

**DOI:** 10.3390/ph19070987

**Published:** 2026-06-26

**Authors:** Meltem Cayci, Burhan Ceylan, Cem Onal

**Affiliations:** 1 Department of Pharmaceutical Toxicology, Faculty of Pharmacy, Harran University, Sanlıurfa 63050, Turkey; 2 Department of Pharmacognosy, Faculty of Pharmacy, Harran University, Sanlıurfa 63050, Turkey; burhanceylan@harran.edu.tr; 3 Department of Analytical Chemistry, Faculty of Pharmacy, Istanbul Health and Technology University, Istanbul 34445, Turkey; cem.onal@istun.edu.tr

**Keywords:** teriflunomide, HPLC-FL, pre-column derivatization, validation, pharmacokinetics

## Abstract

**Background/Objectives**: Teriflunomide is an active metabolite of leflunomide and acts as a selective and reversible inhibitor of dihydroorotate dehydrogenase, a key enzyme in *de novo* pyrimidine biosynthesis. It exhibits immunomodulatory activity by reducing the proliferation of activated T and B lymphocytes and is widely used in the treatment of rheumatoid arthritis and relapsing multiple sclerosis. This study aimed to develop a rapid, accurate, and simple high-performance liquid chromatography (HPLC) method with fluorometric detection for quantifying teriflunomide in human plasma. **Methods**: Plasma samples were prepared by liquid–liquid extraction followed by pre-column derivatization with NBD-Cl. Teriflunomide was derivatized with 4-chloro-7-nitrobenzofurazan (NBD-Cl) and separated using a reversed-phase C18 column (5 µm, 4.6 × 150 mm) at 30 °C with isocratic elution. The mobile phase consisted of acetonitrile and 0.1% orthophosphoric acid (80:20, *v*/*v*) at a flow rate of 1.1 mL/min. Fluorescence detection was performed at λex = 465 nm and λem = 535 nm. The method meets European Medicines Agency (EMA) guidelines for bioanalytical validation and was successfully applied to pharmacokinetic studies, including AUC_0–t_, AUC_0–∞_, C_max_, T_max_, and t_½_. **Results**: Teriflunomide showed a retention time of 2.55 ± 0.01 min. The method exhibited linearity in the range of 0.01–30 ng/mL (r^2^ = 0.9998), with a limit of detection and quantification of 0.003 and 0.01 ng/mL, respectively. The relative standard deviation was 3.27%. **Conclusions**: This work introduces a novel, cost-effective, and highly sensitive HPLC with fluorescence detection (HPLC-FL) method for the determination of teriflunomide in human plasma, providing an efficient alternative to LC-MS/MS for routine pharmacokinetic and bioequivalence studies.

## 1. Introduction

**Figure 1:**
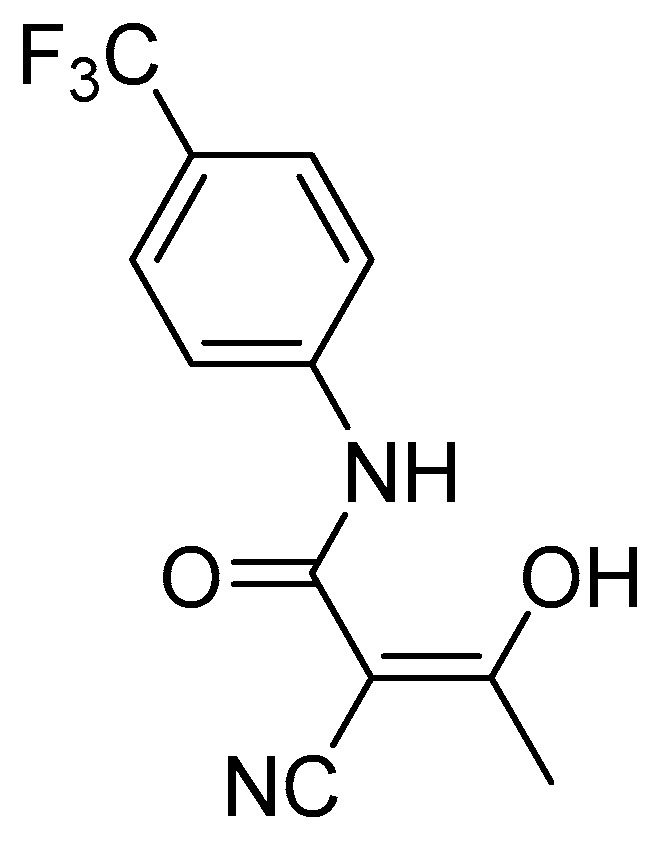
Chemical structure of teriflunomide [5].

Rheumatoid arthritis (RA) is a chronic autoimmune disorder characterized by persistent synovial inflammation, progressive joint destruction, and long-term disability. Disease-modifying antirheumatic drugs (DMARDs) represent the cornerstone of therapy, among which leflunomide is frequently prescribed. Leflunomide is a prodrug that undergoes rapid metabolism to its active metabolite teriflunomide (A77 1726) [1]. Teriflunomide (Figure 1) acts as a selective and reversible inhibitor of dihydroorotate dehydrogenase, an essential enzyme in the de novo pyrimidine biosynthesis pathway, resulting in reduced proliferation of activated T and B lymphocytes [2]. Owing to these immunomodulatory effects, teriflunomide is approved not only as the active metabolite responsible for leflunomide’s clinical activity in RA, but also as a disease-modifying therapy for relapsing forms of multiple sclerosis (MS) [3,4].

Because leflunomide is almost completely converted to teriflunomide, the measurement of teriflunomide levels is more clinically relevant for pharmacokinetic, therapeutic drug monitoring, and bioequivalence studies [6,7]. Consequently, a variety of analytical methods have been reported for the quantification of teriflunomide in pharmaceutical formulations and biological matrices. Early studies employed HPLC with UV detection for the assay of teriflunomide in plasma [8,9,10]. Although these methods were relatively simple, they generally suffered from limited sensitivity and long run times, rendering them less suitable for trace-level monitoring in clinical samples.

To address these limitations, several liquid chromatography-tandem mass spectrometry (LC-MS/MS) approaches were subsequently developed for teriflunomide determination in human plasma, urine, serum and even dried blood spot (DBS) matrices [11,12,13,14,15]. These methods offer excellent selectivity and sensitivity across wide dynamic ranges; however, they require expensive instrumentation, specialized operators, and complex sample preparation steps. More recently, advanced chromatographic strategies such as LC-QTOF-MS [16], UPLC with stability-indicating designs [17,18], and quality-by-design (QbD)-based RP-HPLC protocols [19] have also been reported. These innovations highlight the continuous efforts to enhance sensitivity, robustness, and throughput for teriflunomide quantification.

Despite these advances, there remains a need for a simple, cost-effective, and sensitive analytical alternative that can be implemented in routine pharmaceutical and clinical laboratories without access to sophisticated mass spectrometers. Fluorescence detection, when coupled with appropriate derivatization strategies, provides a promising approach. Among available derivatives, 4-chloro-7-nitrobenzofurazan (NBD-Cl) is widely used due to its ability to form stable, highly fluorescent derivatives with primary and secondary amines. Application of pre-column derivatization with NBD-Cl has been shown to markedly improve sensitivity and selectivity in HPLC assays of various drugs [20,21,22].

To date, no study has reported an HPLC-FL method for the quantification of teriflunomide based on NBD-Cl derivatization. Therefore, the present work aims to develop and validate a novel, reliable, and sensitive HPLC-FL method using pre-column derivatization with NBD-Cl for the determination of teriflunomide. This approach is expected to provide a practical and economical alternative to LC-MS/MS, suitable for routine quality control and pharmacokinetic investigations.

## 2. Results

### 2.1. Derivatization Process

**Figure 2:**
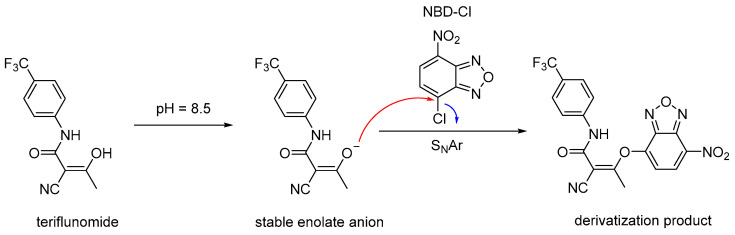
Derivatization reaction between teriflunomide and NBD-Cl [23].

The derivatization conditions between teriflunomide and NBD-Cl were systematically investigated and optimized to enhance the efficiency of the fluorescent reaction product. Each variable was individually adjusted while keeping all other parameters constant to evaluate its specific influence. The optimal values for reaction time, temperature, solution pH, buffer type, acetonitrile-to-water ratio, molar ratio of NBD-Cl to teriflunomide, hydrochloric acid concentration, and the volume required to terminate the derivatization reaction were determined through this approach. The derivatization reaction between teriflunomide and NBD-Cl is presented in Figure 2.

#### 2.1.1. Effect of pH

NBD-Cl is a non-fluorescent labelling reagent that forms intensely fluorescent derivatives upon reaction with functional groups such as primary amines or thiols. It has been widely employed in analytical chemistry due to its simplicity and effectiveness in tagging N-terminal amino acids. In addition, proline-containing peptides can be distinguished based on the distinct fluorescence intensities and emission colors observed following derivatization [24]. In this study, the effect of pH on the reaction was examined within the range of 7 to 11 using phosphate buffer, as the formation of the fluorescent product was found to occur only under alkaline conditions [25].

#### 2.1.2. Effect of Time and Temperature

To determine the optimal conditions for fluorophore formation, the derivatization reaction was carried out at different temperatures and time intervals. Reaction mixtures were incubated in a thermostatically regulated water bath, with temperature settings ranging from 30 °C to 100 °C and reaction durations spanning from 0 to 10 min. The highest fluorescence intensity was achieved by heating the mixture at 80 °C for 7 min, which was selected as the optimal condition for subsequent experiments.

#### 2.1.3. Effect of NBD-Cl Concentration

To determine the appropriate amount of NBD-Cl for derivatizing teriflunomide, various concentrations were tested, ranging from 0.005 to 0.030 mmol. It was observed that derivatization did not occur when the amount of NBD-Cl was below 0.025 mmol, as no corresponding signal appeared in the chromatograms. Once the concentration reached 0.025 mmol (equivalent to 500 µL of a 0.5% *w*/*v* solution), a maximum and stable peak intensity was achieved. Increasing the amount of reagent beyond this level did not lead to any further change in the chromatographic response. Therefore, 0.025 mmol of NBD-Cl was deemed sufficient for complete derivatization of teriflunomide.

#### 2.1.4. Effect of Acetonitrile to Water Ratio in Derivatization Medium

To optimize the solvent composition, different volume ratios of acetonitrile and water were evaluated, keeping the concentrations of teriflunomide, buffers, and NBD-Cl constant. The highest chromatographic response was obtained when the acetonitrile-to-water ratio was adjusted to 1:3 (*v*/*v*).

#### 2.1.5. Stoichiometry of the Reaction

Job’s method of continuous variation was employed to investigate the molar relationship between NBD-Cl and teriflunomide in the reaction medium [26]. By utilizing equimolar concentrations of both compounds, the stoichiometry was found to approximate a 1:1 ratio. The resulting chromatographic peak areas suggested complete consumption of the reagent, with no evidence of excess or deficiency, supporting this stoichiometric conclusion. To establish optimal conditions for the derivatization process, each prepared solution was analyzed by HPLC, and corresponding peak areas were recorded. The formed derivatives demonstrated stability for at least 24 h under these conditions.

#### 2.1.6. Effect of HCl Concentration for Acidification

The optimal outcome was achieved by adding 0.2 mL of 1.0 N HCl, which facilitated the formation of NBD-OH, effectively removing excess NBD-Cl from the reaction mixture.

### 2.2. Chromatographic Process

**Figure 3:**
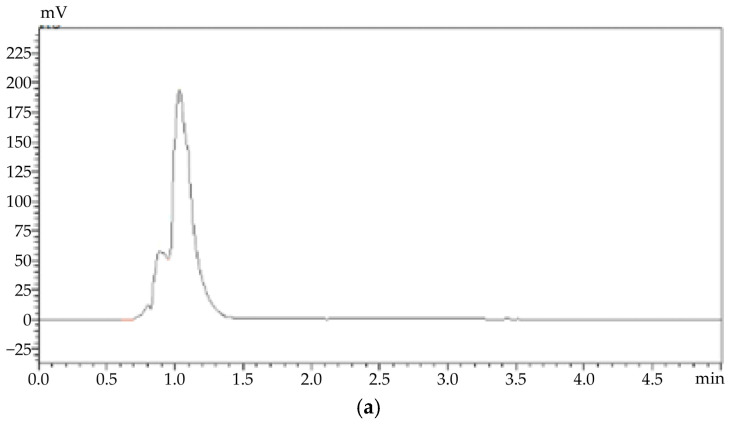

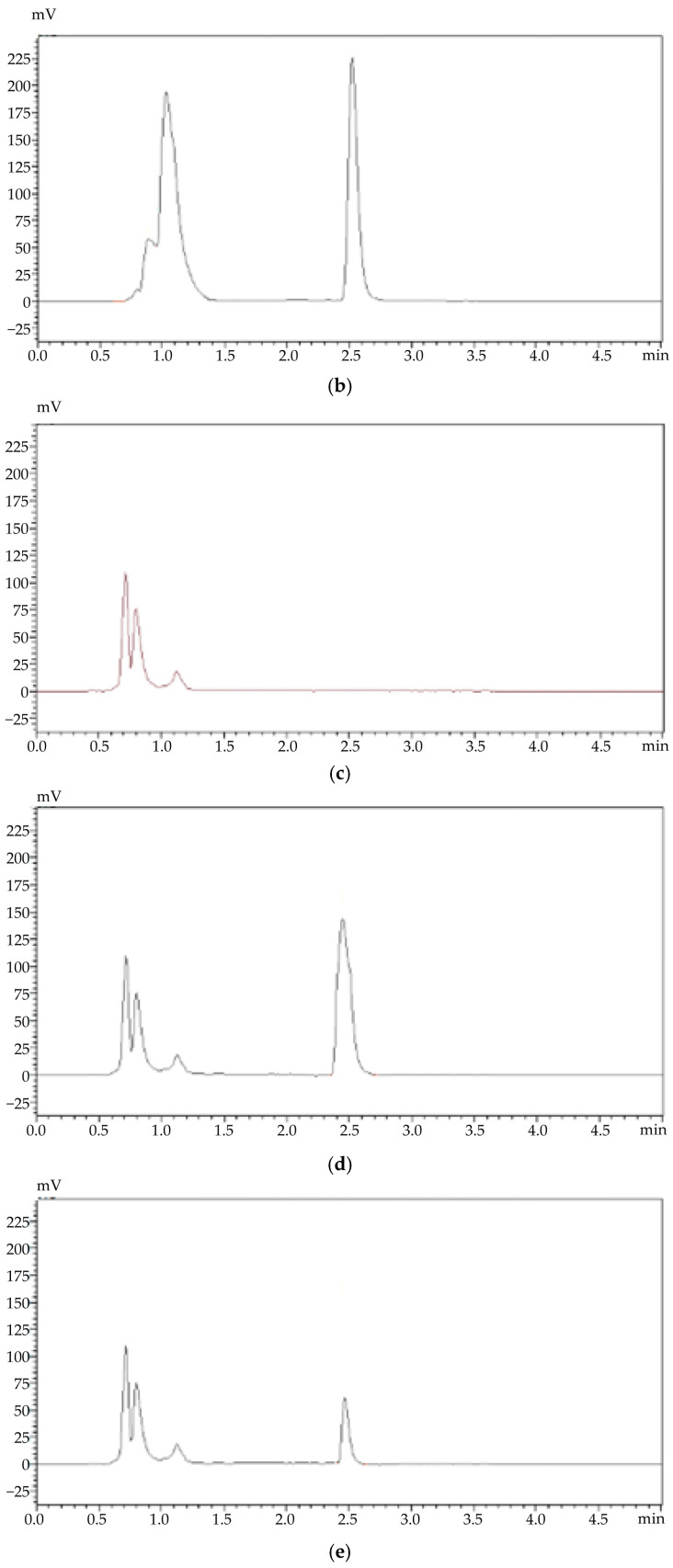
(**a**) blank (aqueous medium), (**b**) standard solution (20 ng/mL standard teriflunomide solution), (**c**) blank plasma sample, (**d**) 10 ng/mL teriflunomide spiked to plasma, (**e**) plasma sample of volunteer after t_max_.

Using an isocratic elution and HPLC-FL, a satisfactory separation of the derivatives and endogenous compounds in plasma was achieved. Figure 3a–e present representative chromatograms of blank plasma, plasma sample spiked with 10 ng/mL teriflunomide, and plasma obtained from a volunteer who had taken a single oral dose of Arava^®^ tablets containing 20 mg of leflunomide at the drug’s maximum plasma concentration time (t_max_).

No interference was observed from endogenous plasma components. Teriflunomide exhibited a retention time of 2.55 ± 0.01 min. Chromatographic system suitability parameters are summarized in Table 1.

### 2.3. Validation of the Method

The analytical method was validated in accordance with the European Medicines Agency (EMA, 2023) [27] and the U.S. Food and Drug Administration (FDA, 2018) [28]. Based on the excellent recovery, accuracy, and precision results obtained during validation, no significant variability attributable to extraction, derivatization, or chromatographic analysis was observed. Therefore, the method was successfully validated without the use of an internal standard. The method was validated by the International Council for Harmanosation (ICH, 2023) [29] bioanalytical method validation guidelines including linearity, accuracy, precision, recovery, selectivity, LOD, LOQ, and stability.

#### 2.3.1. Linearity and Sensitivity

The linearity of the method was evaluated using a calibration curve constructed over a concentration range of 0.01–30 ng/mL of teriflunomide (*n* = 7). Calibration curves were generated by analysing 1.0 mL plasma samples spiked with varying concentrations of teriflunomide. These samples were subjected to the extraction, derivatization, chromatographic separation, and fluorescence detection procedures described in the Materials and Methods section. Calibration curves were constructed by plotting the peak areas of the teriflunomide derivative against the corresponding teriflunomide concentrations using linear least-squares regression analysis. The resulting calibration equation obtained from five calibration levels was: y = 97,968x + 1101.2 (correlation coefficient, r^2^ = 0.9998), where x represents the teriflunomide concentration and y the peak area of the derivatized teriflunomide-NBD-Cl. A summary of the analytical performance parameters of the proposed method is presented in Table 2. The calculated LOD and LOQ values were 0.003 ng/mL and 0.01 ng/mL, respectively.

#### 2.3.2. Accuracy, Precision and Recovery

Quality control (QC) samples were analysed at three concentration levels to evaluate the method’s accuracy and precision. QC samples were prepared in both plasma and aqueous media at low (0.01 ng/mL), medium (5.0 ng/mL), and high (30 ng/mL) concentrations (n = 5). Accuracy was expressed in terms of recovery and relative mean error (RME), while precision was assessed using the relative standard deviation (RSD). To assess the absolute recovery of teriflunomide from plasma, spiked plasma samples were extracted and derivatized, and the resulting peak areas were compared to those obtained from derivatized, unextracted aqueous teriflunomide solutions of the same concentrations. The mean absolute recovery of teriflunomide was found to be 99.71%. The mean relative recovery, determined by comparing the measured concentrations (based on the calibration curve) to the known spiked amounts, was 98.93%.

Intraday precision and accuracy were determined by analyzing three replicates of each concentration on the same day, while interday precision and accuracy were evaluated across three different days. In all cases, the RSD values for both intraday and interday analyses were below 3.27%, indicating high method precision. Overall, these results demonstrate that the method possesses good accuracy and precision, as summarized in Table 3. The recovery study was performed independently from the accuracy and precision assessments.

#### 2.3.3. Robustness

As described in the validation section, the robustness of the method was evaluated by analyzing QC samples at three concentration levels (n = 5). To assess the robustness of the procedure, key chromatographic parameters were deliberately varied, including the flow rate, column oven temperature, and the composition of the mobile phase (acetonitrile-water). Specifically, the column temperature was adjusted from the nominal 30 °C to 25 °C and 35 °C; the acetonitrile-to-water ratio in the mobile phase was altered from 80:20 to 85:15 and 75:25 (*v*/*v*); and the flow rate was varied from 1.1 mL/min to 1.0 and 1.2 mL/min. These modifications did not result in any significant changes in peak area or resolution. The consistently low RSD values observed confirm the robustness of the method, as summarized in Table 4.

#### 2.3.4. Stability

The stability of working standard solutions of teriflunomide at QC levels was evaluated using three replicated under various storage conditions. The tested conditions included storage at room temperature in the dark for 24 h, autosampler conditions for 24 h, and refrigeration at 4 °C for one month. The recovery rates observed under these conditions were 98.4%, 98.1%, and 99.2%, respectively. These values were higher than those reported in previous studies. The highest relative standard deviation (RSD) observed among all tests was 3.43%, indicating good consistency. These results demonstrate that teriflunomide is stable under all the tested storage conditions.

### 2.4. Method Applicability Demonstration in Human Plasma

The proposed method was applied to determine teriflunomide concentrations in plasma for pharmacokinetic evaluation. A single oral dose of (Arava^®^) tablet containing 20 mg leflunomide was administered to a healthy female volunteer aged 55. This pilot pharmacokinetic evaluation was conducted on a single healthy volunteer to demonstrate the applicability of the developed method. On the first day, approximately 5 mL of venous blood were collected prior to dosing and at 0.25, 0.5, 0.75, 1, 2, 3, 4, 5, 6, 7, 8 and 12 h post-dose. As previously described, the blood samples were processed to obtain plasma and stored at −20 °C until analysis.

**Figure 4:**
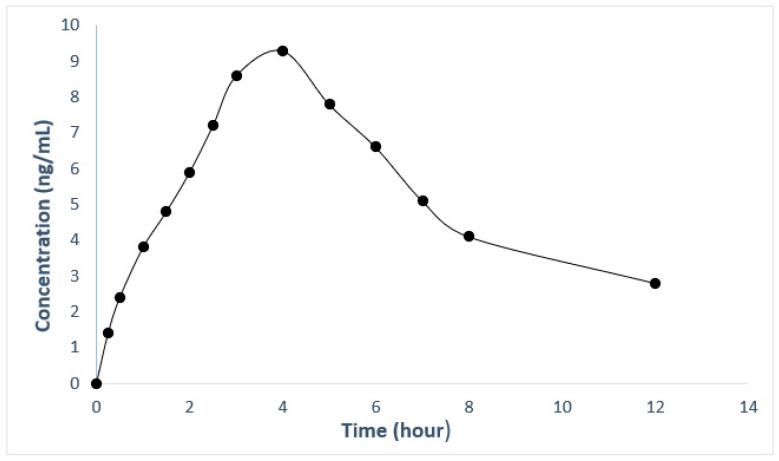
Pharmacokinetic curve of teriflunomide after administration of 20 mg dose orally.

A representative chromatogram of the plasma sample collected 2.0 h after oral administration of 20 mg teriflunomide is shown in Figure 3e. Pharmacokinetic parameters were calculated based on the plasma concentrations obtained using the validated analytical method. The TOPFIT 2.0 pharmacokinetic and pharmacodynamics data analysis software was employed to compute the area under the plasma concentration–time curve (AUC_0–12_, AUC_0–∞_) [30]. The plasma concentration–time profile of teriflunomide following administration of a single 20 mg oral dose is illustrated in Figure 4. The pharmacokinetic data presented in Table 5 are provided solely to demonstrate the applicability of the developed method to human plasma samples and should be regarded as preliminary results from a pilot proof-of-concept application [31].

## 3. Discussion

In the present study, a novel HPLC-FL method based on pre-column derivatization with NBD-Cl was successfully developed and validated for the determination of teriflunomide in human plasma. Compared with previously reported HPLC-UV and spectrophotometric methods, the proposed assay demonstrated superior sensitivity and shorter analysis time. The method showed excellent linearity over the concentration range of 0.01–30 ng/mL with a correlation coefficient of 0.9998. The obtained LOD and LOQ values were 0.003 ng/mL and 0.01 ng/mL, respectively, which are markedly lower than those reported for UV spectrophotometric methods (LOD: 0.25 µg/mL; LOQ: 0.625 µg/mL).

Several LC-MS/MS methods have been reported for teriflunomide quantification in biological matrices with linear ranges generally between 10–5000 ng/mL and run time around 3 min. Although these methods provide high sensitivity and selectivity, they require sophisticated instrumentation and higher operational costs. In contrast, the proposed HPLC-FL method achieved comparable rapid separation with a retention time of 2.55 min using simpler and more accessible instrumentation. The human plasma application was intended solely as a proof-of-concept demonstration of method applicability. Therefore, no conclusions regarding pharmacokinetic behavior, bioavailability, or bioequivalence should be drawn from these data. Further studies involving larger subject populations would be required for comprehensive pharmacokinetic evaluation.

Optimization studies demonstrated that maximum fluorescence intensity was obtained at pH 8.5 after heating at 80 °C for 7 min using 0.025 mmol NBD-Cl. The derivatization product remained stable for at least 24 h. The method also exhibited excellent extraction efficiency, with mean absolute and relative recovery values of 99.71% and 98.93%, respectively. Precision studies showed that intraday and interday RSD values were below 3.27%, confirming good reproducibility in accordance with EMA and FDA validation criteria [27,28].

Robustness testing revealed that small variations in flow rate, mobile phase composition, and column temperature did not significantly affect chromatographic performance, with recovery values remaining above 95%. Furthermore, the developed method was successfully applied to a pilot pharmacokinetic study following oral administration of 20 mg teriflunomide.

Overall, the proposed HPLC-FL method provides a rapid, sensitive, and cost-effective alternative to LC-MS/MS for routine teriflunomide analysis in human plasma and may be suitable for pharmacokinetic, bioavailability, and bioequivalence studies.

## 4. Materials and Methods

### 4.1. Chemicals and Reagents

Teriflunomide was obtained from Shanghai Yingxuan Pharmaceutical Science & Technology (Shi, China), while Arava^®^ film-coated tablets containing 20 mg of leflunomide were procured from a licensed local pharmacy. Reagents and solvents used in the study included HPLC-grade acetonitrile and orthophosphoric acid, analytical-grade dimethyl sulfoxide, methanol and chloroform, as well as monobasic and dibasic phosphate salts and hydrochloric acid, all supplied by Merck (Darmstadt, Germany). The derivatizing agent NBD-Cl was sourced from Sigma Aldrich (St. Louis, MO, USA). Ultrapure water was produced using a Human-brand water purification system (Shi, China).

### 4.2. Instrumentation and Chromatographic Conditions

Fluorescence data were acquired using a Shimadzu RF-1501 spectrofluorometer (Shimadzu Corporation, Kyoto, Japan) equipped with a xenon arc lamp and standard 1 cm quartz cuvettes. The optimal excitation and emission wavelengths for the teriflunomide-NBD derivative were established at 465 nm and 535 nm, respectively. pH values during the procedure were monitored using a WTW model 526 digital pH meter. Chromatographic analyses were carried out using a Shimadzu LC-20A series HPLC system (Shimadzu Corporation, Kyoto, Japan) equipped with a fluorescence detector. Various chromatographic conditions were systematically tested to optimize the separation parameters. The final method utilized an Inertsil^®^ C18 (150 × 4.6 mm, 5 µm particle size) column (Shimadzu Corporation, Kyoto, Japan) maintained at 30 °C. Isocratic elution was achieved using a mixture of acetonitrile and 0.1% orthophosphoric acid in water (80:20, *v*/*v*) at a flow rate of 1.1 mL/min.

### 4.3. Solutions

Standard working solutions ranging from 0.01 to 30 ng/mL were prepared by serial dilution of a teriflunomide stock solution (0.1 mg/mL) in dimethyl sulfoxide. A phosphate-buffered solution was prepared by dissolving 2.0209 g of disodium hydrogen phosphate and 0.3394 g of monosodium dihydrogen phosphate in 50 mL of distilled water. The pH was adjusted to 8.5 using 0.1 M hydrochloric acid, and the final volume was brought up to 100 mL with ultrapure water. A fresh solution of NBD-Cl was prepared in methanol at a concentration of 5 mg/mL. All prepared solutions, except for NBD-Cl, remained chemically stable for at least two weeks when stored at 4 °C.

### 4.4. Limit of Detection (LOD) and Limit of Quantitation LOQ)

The limit of detection (LOD) and limit of quantification (LOQ) were calculated using the formula LOD or LOQ = kSDa/b, where k = 3 for LOD and k = 10 for LOQ, SDa is the standard deviation of the intercept, and b is the slope of the calibration curve.

### 4.5. Sample Preparation and General Procedure

Approximately 5 mL of venous blood was collected from a healthy volunteer via peripheral venipuncture following ethical approval and informed consent. Blood samples were transferred into tubes containing disodium EDTA as an anticoagulant and centrifuged at 4500× *g* for 10 min to separate plasma. The plasma aliquots obtained were stored at −20 °C until analysis.

To extract teriflunomide from plasma, 1.0 mL plasma was first basified with 500 µL of 0.1 M sodium hydroxide. Following this, appropriate volumes of teriflunomide working standard solutions were added. The mixture was then subjected to liquid–liquid extraction using 5 mL of chloroform. After vortexing the mixture at moderate speed for 5 min, it was centrifuged again at 4500× *g* for 5 min. The aqueous (upper) layer was carefully discarded.

The organic phase was transferred to a clean tube and evaporated to dryness under a gentle stream of nitrogen at 40 °C. The resulting residue was reconstituted with 500 µL of phosphate buffer (pH 8.5), 500 µL of NBD-Cl solution (5 mg/mL in methanol), and 1 mL of distilled water. This mixture was incubated at 80 °C for 7 min to complete the derivatization reaction. After rapid cooling in an ice bath, 0.2 mL of 1 N HCl was added to acidify the reaction medium. The sample was vortexed vigorously for 30 s, and a 20 µL aliquot of the resulting derivatized solution was injected into the HPLC system for analysis.

## 5. Conclusions

Teriflunomide is an oral immunomodulatory agent widely used in the treatment of relapsing forms of multiple sclerosis (MS). Due to its long-term use and potential for drug–drug and drug–food interactions, as well as adverse effects, the development of sensitive and reliable analytical methods for its quantification in biological matrices is essential. In this study, a simple, cost-effective, and reproducible HPLC-FL method was developed and validated for the determination of teriflunomide in plasma. The method is based on the derivatization of teriflunomide with NBD-Cl, forming highly fluorescent derivatives that enable fluorescence detection. The retention time of the analyte is approximately 2.55 min, indicating a short overall analysis time. To the best of our knowledge, this is the first report in the literature describing the fluorescence detection of teriflunomide using HPLC-FL. Due to its simplicity and high sensitivity, the proposed method can be easily implemented in routine laboratory analyses. Furthermore, the method demonstrated successful applicability to human plasma samples and may be considered for future pharmacokinetic, bioavailability, and bioequivalence studies following appropriate clinical validation.

## Figures and Tables

**Table 1:** Chromatographic system suitability parameters.

Capacity Factor ^1^	Resolution ^1^	HETP (µm) ^1^	Tailing Factor ^1^	Asymmetry Factor ^1^
7.88	2.25	0.08	1.2	1.2

^1^ mean values of the parameters of all the points in calibration study are mentioned.

**Table 2:** Analytical parameters of the method.

Parameters	Method
Concentration range ^a^ (ng/mL)	0.01–30
Regression equation ^b^	y = 97,968x +1101.2
Slope ± SD	97,968 ± 284
Intercept ± SD	1101.2 ± 83.7
Correlation coefficient (r^2^)	0.9998
LOD (ng/mL)	0.003
LOQ (ng/mL)	0.01

^a^ Average of six determinations. ^b^ y = xC + b where C is the concentration in ng/mL and y is the peak area.

**Table 3:** Accuracy and precision of the method.

Existing Concentration (ng/mL)	Added Concentration (ng/mL)	Found Concentration (ng/mL) (Mean ± SD ^1^)	Recovery (%)	RSD of Recovery	RSD of Intraday Variation	RSD of Interday Variation
5 5 5	0.01 5 30	4.93 ± 0.06 9.87 ± 0.03 34.90 ± 0.01	98.40 98.70 99.71	1.08 1.01 0.84	1.34 1.28 1.22	3.27 3.18 3.10
Mean relative recovery = 98.93						

Repeatability 5 for all QC sample levels.

**Table 4:** Robustness of the method.

Condition	Value	Recovery %	RSD %
Flow rate mL/min	1.0 1.2	95.2 95.8	3.89 3.86
Mobile phase composition (acetonitrile: aqueous phase)	75:25 85:15	97.3 97.7	2.27 2.20
Column temperature	25 35	99.1 99.7	0.96 1.03

Repeatability 5 for all QC sample levels.

**Table 5:** Pharmacokinetic parameters of teriflunomide after administration of single oral dose of 20 mg.

Parameter	Found Value
T_max_ ^a^ (h)	4.0
C_max_ ^b^ (ng/mL)	9.3
t_1/2_ ^c^ (h)	8.2
AUC_0–12_ ^d^ (ng.h/mL)	125.3
AUC_0–∞_ ^d^ (ng.h/mL)	148.7

^a^ Time to maximum concentration. ^b^ Maximum concentration. ^c^ Elimination half life. ^d^ Area under the concentration–time curve.

## Data Availability

The original contributions presented in this study are included in the article. Further inquiries can be directed to the corresponding authors.
